# The Development of Models Based on Linear and Nonlinear Multivariate Methods to Predict ADME/PK Properties Using Physicochemical Properties of Kinase, Protease Inhibitors, and GPCR Antagonists

**DOI:** 10.1155/2013/495134

**Published:** 2013-03-19

**Authors:** Deepu Bakasta, M. G. Shambhu

**Affiliations:** ^1^Department of Biotechnology, PES Institute of Technology, Bangalore 560068, India; ^2^Department of Biotechnology, The Oxford College of Engineering, Bangalore 560068, India

## Abstract

Oral bioavailability of a drug compound is the significant property for potential drug candidates. Measuring this property can be costly and time-consuming. Quantitative structure-property relationships (QSPRs) are used to estimate the percentage of oral bioavailability, and they are an attractive alternative to experimental measurements. A data set of 217 drug and drug-like compounds with measured values of the percentage of oral bioavailability taken from the small molecule ChemBioBase database was used to develop and test a QSPR model. Descriptors were calculated for the compounds using Codessa 2.1 tool. Nonlinear general regression neural network model was generated using the DTREG predictive modeling program software. The calculated percentage of oral bioavailability model performs well, with root-mean-square (rms) errors of 4.55% oral bioavailability units for the training set, 14.32% oral bioavailability units for the test set, and 19.12% oral bioavailability units for the external prediction set. Given the structural diversity and bias of the data set, this is a good first attempt at modeling oral bioavailability using QSPR methods. The model can be used as a potential virtual screen or property estimator. With a larger data supply less biased toward the high end values of the percentage of oral bioavailability, a more successful model could likely be developed.

## 1. Introduction

Rational drug discovery requires early methods of all factors influencing on the likely success of a drug candidate in the subsequent stages of the drug development. The study of absorption, distribution, metabolism, excretion, and pharmacokinetics (ADME/PK) has become a major discipline in drug discovery through the application of well-established in vitro and in vivo methodologies [1]. PK plays a crucial role in the pharmaceutical research and development and, because of the major role of drug metabolism, drug discovery research in this area is covered by groups coalesced around the name drug metabolism and pharmacokinetics (DMPKs) [2–6]. Elimination is the product of metabolism and excretion. Pharmacokinetics describe how the body reacts to a specific drug after administration. Pharmacokinetic properties of drugs may be affected by factors such as the administration site and the administered drug dose; these may influence the rate of absorption. One more process, liberation, plays an important role in pharmacokinetics: liberation means the release of drug from the formulation [7].

The primary goal of the drug discovery and development process is to find a molecule possessing both good pharmacodynamics and good pharmacokinetic properties [8]. Ideally, a new drug should be efficacious and selective, target tissue(s) specific, and orally absorbed, cause minimal or no adverse effects due to metabolite activity or toxicity, and be distributed/excreted in such a fashion as to permit dosage once a day. Successful optimization of all these properties is an extremely challenging task. QSPR methods have successfully been used to model physicochemical properties of organic compounds. Several computational models have also been reported for such biopharmaceutical properties as %HIA, blood-brain barrier, skin and ocular permeation, pharmacokinetics and metabolism. However, all these studies involved sets of closely related structural analogues, and models based on limited chemical space generally lack predictive value outside their structural classes. Broadly applicable QSPR models of biopharmaceutical properties must be built using compounds which cover both a wide range of the property being modeled as well as of the chemical structure space [9].

Bioavailability is a key pharmacokinetic parameter which expresses the proportion of a drug administered by any nonvascular route that gains access to the systemic circulation [10]. It was found that 30% of drugs fail during the drug discovery process. Therefore, there is a need of a robust and accurate computational model (QSAR/QSPR) which can predict the oral bioavailability of compounds without carrying out any experiments.

## 2. Materials and Methods

Development of model using linear and nonlinear multivariate statistics to predict the oral bioavailability of a compound.

### 2.1. Quantitative Structure Property Relationship

The QSPR methodology used in this project consists of three main parts: representation of molecular structure, feature selection, and mapping. The general assumption in QSPR modeling is that molecular structure causes the observed behavior of a compound, linking a series of chemical structures to properties of interest; in this case, oral bioavailability should provide a method for modeling the property. General regression neural network method present in DTREG was used to generate the oral bioavailability model. This neural network has 3 layers and 1 hidden layer, and it uses kernel function.

### 2.2. Preparation of Data Set

The set of 217 drug and drug-like compounds and their experimentally derived percentage of oral bioavailability values used in this project were gathered from the literature sources. The 217 compounds in the working data set were classified by using sphere exclusion algorithm [11] into a training set of 159 compounds, test set of 50 compounds, and an external prediction set of 8 compounds. The external prediction set was chosen in such a way as to cover the range of percentage of oral bioavailability values in the data set, and it spans the range of 0%–100%. The compounds in the external prediction set were never used during the model development process but were reserved to validate potential models. The structures of the 217 compounds were extracted from the small molecule ChemBioBase [12] database with ISIS which consists of kinase, protease inhibitors, and GPCR antagonists. Accurate geometries are necessary for the calculation of certain descriptors thought to be necessary for modeling physical and chemical properties. As a result, LigPrep [13] tool was used to generate accurate three-dimensional geometries, and conformational analysis was performed in order to get the local minima structure which is necessary for calculating geometric descriptors.

### 2.3. Descriptor Generation and Analysis

A total of 11 descriptors were generated for each of the 217 compounds using Codessa 2.1 tool [14]. The descriptors fall into three general categories: topological, electronic, and geometric. Topological descriptors like number of single bonds, number of aromatic bonds, number of oxygen atoms, nitrogen atoms, relative number of oxygen atoms, and relative number of nitrogen atoms are derived from the information of the two-dimensional structure of the molecule.

Electronic descriptors were calculated by MOPAC using the AM1 Hamiltonian electronic descriptors include maximum partial charge for hydrogen atoms. Geometric descriptors include cube root of gravitational index, SAAA (surface area of hydrogen bond acceptor atoms/number of hydrogen bond acceptor atoms), and normalized 2D projection on YZ plane. Accurate three-dimensional geometries of the molecules are necessary to calculate descriptors of this nature. A fourth class of descriptors can be derived by combining electronic and geometric information to form hybrid descriptors. By combining the molecular surface area with partial atomic charges, charged-partial surface area (CPSA) descriptors can be calculated. This includes HASA-2, area-weighted surface charge of hydrogen bonding acceptor atoms. Eleven descriptors span the following ranges: number of single bonds (26–90), number of aromatic bonds (0–12), YZ shadow (29.9–110.46), GRAV-3 (25.97–42.16), SAAA (28.38–32.14), number of oxygen atoms (0–7), number of nitrogen atoms (1–8), relative number of oxygen atoms (0–0.12), relative number of nitrogen atoms (0.02–0.17), maximum partial charge for a hydrogen atom (0.03–0.1), and HASA-2 (2.89–32.2). First two descriptors indicate amount of structural flexibility; next two descriptors encode molecular size, shape, and bulk properties. Remaining descriptors are all hydrogen bonding descriptors.

### 2.4. Descriptors

1. Topological descriptors include number of single bonds, number of oxygen atoms, relative number of oxygen atoms, number of nitrogen atoms, relative number of nitrogen atoms, and number of aromatic bonds [15].
2. Geometric descriptors include the following:
  - GRAV-3: cube root of gravitational index [16],
  - SAAA-2: surface area of hydrogen bond acceptor atoms/number of hydrogen bond acceptor atoms; these two are calculated using java program,
  - SHDW-6: normalized 2D projection of molecule on YZ plane [16],
3. maximum partial charge for hydrogen atoms [17],
4. HASA-2: area-weighted surface charge of hydrogen bonding acceptor atoms [18].

The NSB descriptor is encoding single bonds, and this may be an indication of the amount of structural flexibility [19]. The SHDW-6 and GRAV-3 descriptors are encoding molecular size, shape, and bulk properties [17]. These size descriptors may be important with respect to the ability of the drug to penetrate cell membranes. The three remaining descriptors are all hydrogen bonding descriptors (Table 2). 

## 3. Results and Discussion

### 3.1. Absorption Ellipse Model

**Figure 1:**
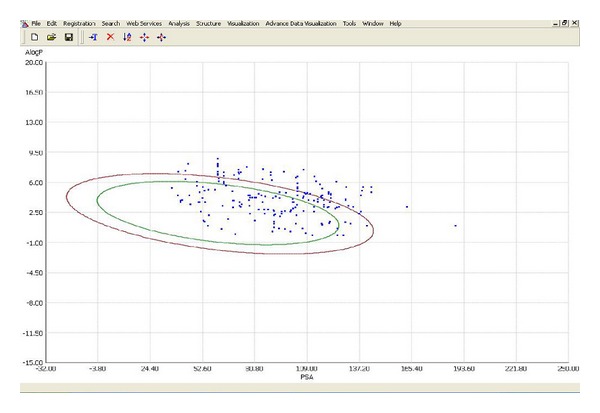
AlogP versus PSA.

In this model, Alogp and PSA descriptors were used as predictor variables. Well-absorbed and poor-absorbed compounds were plotted against AlogP versus PSA. A 95% confidence ellipse for the well-absorbed dataset was also computed and plotted. The 95% confidence ellipse represents the region of chemical space where we can expect to find well-absorbed compounds (90%) 95 out of 100 times (Figure 1). 

CIMPL 1.0 [19] version tool was used to generate 95% confidence ellipse model. It gives more than 60% of misclassification; from this, we conclude that the Alogp and PSA (polar surface area) descriptors are not enough for this dataset to classify well-absorbed and poor-absorbed molecules. Calc prop tool was used to calculate Alogp and PSA. 95% confidence means that we get 95 times out of 100 molecules having good absorption inside the ellipse. So, it is called 95% confidence ellipse.

**Figure 2:**
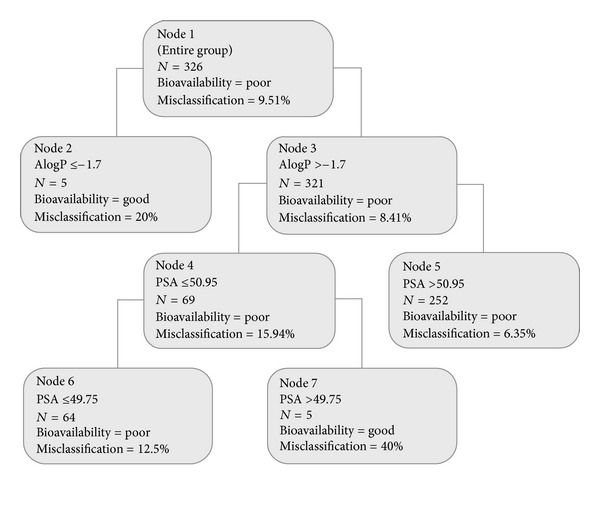
Single decision tree method.

DTREG [20] software was used to generate single decision tree (Figure 2); this model also gives a lot of misclassification (more than 40%). Both absorption ellipse method and single decision tree methods show a lot of misclassification. Alogp and PSA descriptors are not enough to classify well-absorbed and poor compounds.

### 3.2. General Regression Neural Network Method (DTREG Software)

Partial least square method and multiple linear regression present in CIMPL tool were employed to get good linear models; none of the models found was satisfactory. It became evident that the diversity of this data set and the number of data points possessing greater than 50% absorption values are largely responsible for producing poor quality linear models. Therefore, this data set became a good candidate for developing nonlinear neural network model.

### 3.3. Neural Network Analysis

**Figure 3:**
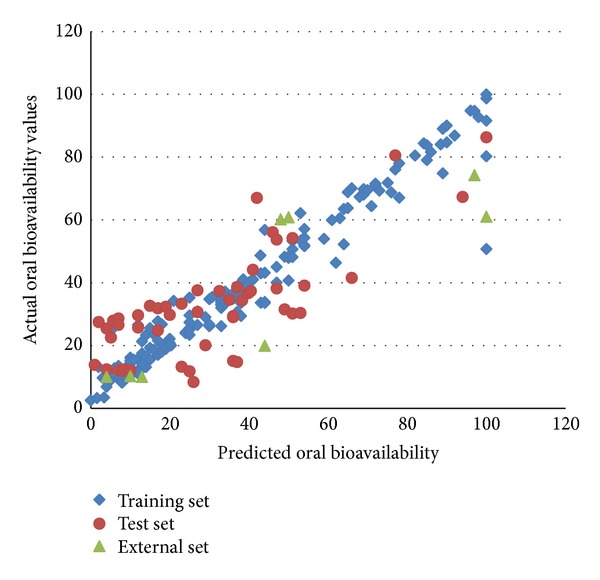
Actual percentage of oral bioavailability values versus predicted values.

**Figure 4:**
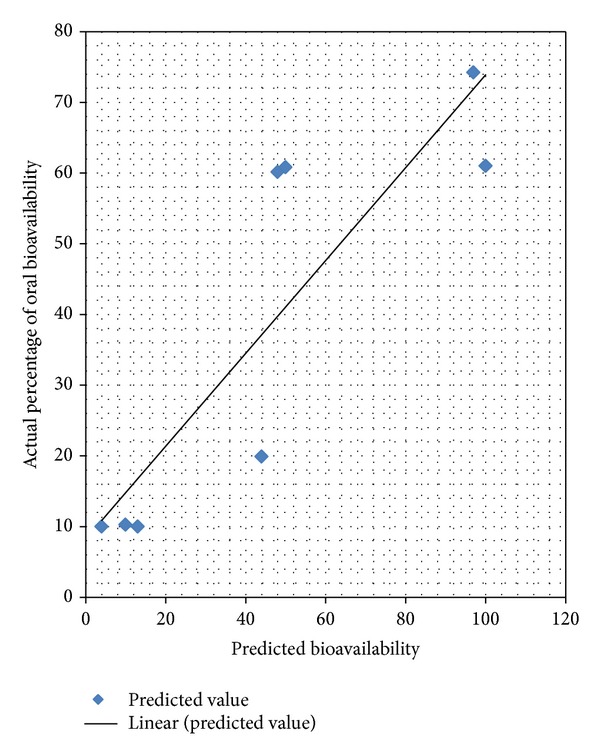
Actual percentage of oral bioavailability values versus predicted values (external prediction set).

11-member reduced pool of descriptors was fed to the general regression neural network method for the purpose of developing a nonlinear model. The original regression data set was split randomly into a neural network training set of 159 compounds and a test set of 50 compounds using sphere exclusion method. The original 8-member external prediction set was used to validate any neural network models. The test set was used to monitor overtraining of the network, and the training set was used to actually train the network (Figures 3 and 4). 

To decrease the possibility of chance effects influencing neural network training, the ratio of observations to total adjustable parameters should be at or above 2.0. A neural network consisting of 11 input neurons (descriptors), 3 hidden neurons, and 1 output neuron (target and percentage of bioavailability), thus producing an 11-3-1 architecture, was used since it produced the maximum number of adjustable parameters recommended for a dataset of this size. For this 11-3-1 architecture, the ratio of training set observations to adjustable parameters *F* was 67/33 or 2.03 (Table 1). Table 1 refers to statistics results of *R* ^2^, RMSE, MAE, and standard deviation for training set, test set, external prediction set.

## 4. Conclusion

11-descriptor general regression neural network model has been developed for the estimation of the percentage of oral bioavailability values for a data set of 217 drug and drug-like compounds. The training set rms error was 4.55% oral bioavailability units, and the test set rms error was 14.32% oral bioavailability units. Based on the rms errors of the training and test sets, it is clear that a link between the structure and the percentage of oral bioavailability does exist. However, the strength of that link is best measured by the quality of the external prediction set. With rms error of 19.0% HIA units and a good visual plot, the external prediction set ensures the quality of the model. Given the structural diversity and bias of the data set, this is a good first attempt at modeling oral bioavailability using QSPR methods. The optimization of individual properties along the absorption process must be integrated in a multiobjective scenario for studying oral bioavailability behavior in the early drug discovery and development [21].

The model can be used as a potential virtual screen or property estimator. With a larger data supply less biased toward the high end values of the percentage of oral bioavailability, a more successful and reviling model can be developed. This study illustrates the potential of using QSPR methods to aid in the drug development process.

## Supplementary Material

Table 1 represents supplementary data for training data and test data.
Table 2 represents supplementary data for A Total of 11descriptors were generated for each of the 217 compounds using Codessa 2.1 tool.

## Figures and Tables

**Table 1:** Statistics results of *R* ^2^, RMSE, MAE, and standard deviation for training set, test set, external prediction set.

	*R* ^2^	RMSE	MAE	Standard deviation
Training set	0.98	4.55	2.90	37.68
Test set	0.77	14.32	12.16	22.64
External prediction set	0.86	19.14	14.76	37.20

**Table 2:** Correlation matrix for descriptors.

Variable	G. index	S. bonds	SAAA	YZ shadow	HASA-2	P. charge	O. atoms	R. O. atoms	N. atoms	R. N. atoms	A. bonds
G. index	1										
S. bonds	0.26798	1									
SAAA	0.41483	−0.0544	1								
YZ shadow	0.50115	0.31235	0.21755	1							
HASA-2	0.13998	−0.05302	−0.02074	0.18598	1						
P. charge	−0.03232	−0.1199	0.03092	0.03528	0.30933	1					
O. atoms	0.11838	0.09889	0.04789	−0.00181	0.12292	0.13269	1				
R. O. atoms	−0.02622	−0.31383	0.05442	−0.14418	0.11698	0.16337	0.58024	1			
N. atoms	0.21829	0.27275	0.02458	0.21158	0.50736	−0.10747	−0.11228	−0.29378	1		
R. N. atoms	0.02254	−0.32032	0.05143	−0.01294	0.43585	−-0.10581	−0.24878	−0.1698	0.52116	1	
A. bonds	0.2846	0.2069	0.00138	0.33825	0.14602	0.159	0.01585	−0.1204	−0.02972	−0.24046	1

## Abbreviations

CIMPL: Chemo informatics platform tool
ADME/Tox: Absorption, distribution, metabolism, excretion, and toxicity
QSPR: Quantitative structure property relationship
HIA: Human intestinal absorption
PSA: Polar surface area
RMSE: Root mean square error
MAE: Mean absolute error
IV: Intravenous.
