# Supplementary material for: The Development of Models Based on Linear and Nonlinear Multivariate Methods to Predict ADME/PK Properties Using Physicochemical Properties of Kinase, Protease Inhibitors, and GPCR Antagonists
Source: Int J Med Chem. 2013 Mar 19;2013:495134. doi: 10.1155/2013/495134 (PMC4207418; doi:10.1155/2013/495134)
Supplement: Supplementary file 1 — Table 1 represents supplementary data for training data and test data. Table 2 represents supplementary data for A Total of 11descriptors were generated for each of the 217 compounds using Codessa 2.1 tool. [file 495134.f1.docx]

Supplementary materials

Table-1 Represents supplementary data for training and test data

| **s.no componuds** | **Train/test set** | **BA** | **logit** | **des-01** | **des-02** | **des-03** | **des-04** | **des-05** | **des-06** | **des-07** | **des-08** | **des-09** | **des-10** | **des-11** |
| --- | --- | --- | --- | --- | --- | --- | --- | --- | --- | --- | --- | --- | --- | --- |
| str-001 | Tr | 0 | -1.28 | 29.75 | 38 | 29.96 | 39.98 | 11.19 | 0.10 | 1 | 0.02 | 4 | 0.09 | 3 |
| str-002 | Ts | 1 | -1.20 | 35.49 | 74 | 29.68 | 71.64 | 14.12 | 0.06 | 4 | 0.05 | 5 | 0.06 | 6 |
| str-003 | Tr | 1.6 | -1.16 | 33.55 | 53 | 29.38 | 67.84 | 24.24 | 0.08 | 3 | 0.05 | 4 | 0.06 | 6 |
| str-004 | Ts | 2 | -1.14 | 34.33 | 40 | 29.69 | 77.98 | 15.72 | 0.06 | 3 | 0.06 | 4 | 0.08 | 6 |
| str-005 | Tr | 2 | -1.14 | 33.02 | 44 | 29.55 | 58.44 | 23.58 | 0.06 | 5 | 0.10 | 4 | 0.08 | 3 |
| str-006 | Tr | 3 | -1.08 | 37.16 | 77 | 29.05 | 94.56 | 15.32 | 0.06 | 4 | 0.05 | 5 | 0.06 | 6 |
| str-007 | Tr | 3.4 | -1.06 | 33.98 | 54 | 29.34 | 69.82 | 26.11 | 0.10 | 4 | 0.06 | 4 | 0.06 | 6 |
| str-008 | Ts | 4 | -1.03 | 36.34 | 61 | 29.28 | 80.58 | 24.71 | 0.06 | 5 | 0.07 | 6 | 0.08 | 7 |
| str-009 | Ts | 4 | -1.03 | 37.04 | 86 | 29.73 | 81.52 | 11.95 | 0.05 | 1 | 0.01 | 4 | 0.04 | 6 |
| str-010 | Tr | 4 | -1.03 | 37.60 | 61 | 29.96 | 97.24 | 19.40 | 0.06 | 4 | 0.05 | 6 | 0.08 | 3 |
| str-011 | Tr | 5 | -0.98 | 37.49 | 79 | 29.69 | 75.58 | 15.25 | 0.06 | 4 | 0.04 | 5 | 0.06 | 6 |
| str-012 | Tr | 5 | -0.98 | 36.30 | 81 | 29.68 | 82.84 | 18.93 | 0.06 | 2 | 0.02 | 5 | 0.05 | 9 |
| str-013 | Tr | 5 | -0.98 | 37.50 | 67 | 29.86 | 97.32 | 12.50 | 0.04 | 3 | 0.04 | 4 | 0.05 | 7 |
| str-014 | Tr | 5.1 | -0.97 | 35.42 | 66 | 29.76 | 75.16 | 19.07 | 0.05 | 3 | 0.04 | 5 | 0.07 | 6 |
| str-015 | Ts | 5.1 | -0.97 | 35.42 | 66 | 29.76 | 70.54 | 18.81 | 0.05 | 3 | 0.04 | 5 | 0.07 | 6 |
| str-016 | Ts | 5.7 | -0.95 | 37.63 | 68 | 30.78 | 84.36 | 18.88 | 0.08 | 4 | 0.05 | 5 | 0.06 | 6 |
| str-017 | Tr | 6 | -0.94 | 39.71 | 81 | 30.62 | 98.54 | 12.14 | 0.07 | 5 | 0.05 | 5 | 0.05 | 5 |
| str-018 | Tr | 7 | -0.89 | 32.51 | 68 | 29.90 | 56.96 | 20.18 | 0.08 | 4 | 0.05 | 5 | 0.06 | 6 |
| str-019 | Ts | 7 | -0.89 | 37.63 | 84 | 30.78 | 71.16 | 13.23 | 0.05 | 1 | 0.01 | 4 | 0.04 | 6 |
| str-020 | Ts | 7 | -0.89 | 37.63 | 68 | 30.78 | 74.82 | 20.37 | 0.08 | 4 | 0.05 | 5 | 0.06 | 6 |
| str-021 | Tr | 7 | -0.89 | 36.63 | 37 | 29.96 | 87.90 | 13.83 | 0.05 | 1 | 0.02 | 3 | 0.06 | 7 |
| str-022 | Ts | 7 | -0.89 | 37.67 | 65 | 30.78 | 80.20 | 20.62 | 0.08 | 4 | 0.05 | 5 | 0.06 | 6 |
| str-023 | Tr | 7.3 | -0.88 | 34.84 | 49 | 30.03 | 72.64 | 13.43 | 0.03 | 1 | 0.02 | 6 | 0.10 | 5 |
| str-024 | Tr | 7.9 | -0.86 | 36.47 | 78 | 29.52 | 86.90 | 14.91 | 0.10 | 2 | 0.02 | 5 | 0.06 | 6 |
| str-025 | Tr | 8 | -0.86 | 37.04 | 56 | 28.99 | 81.78 | 28.04 | 0.06 | 7 | 0.10 | 5 | 0.07 | 6 |
| str-026 | Ts | 8 | -0.86 | 36.55 | 62 | 29.52 | 73.22 | 20.75 | 0.03 | 2 | 0.03 | 3 | 0.04 | 9 |
| str-027 | Tr | 8 | -0.86 | 35.23 | 77 | 29.73 | 80.40 | 5.03 | 0.04 | 2 | 0.02 | 3 | 0.03 | 8 |
| str-028 | Ts | 8.5 | -0.84 | 35.92 | 75 | 29.96 | 95.42 | 13.19 | 0.05 | 1 | 0.01 | 4 | 0.05 | 6 |
| str-029 | Ts | 9 | -0.82 | 36.48 | 70 | 30.66 | 66.62 | 11.77 | 0.05 | 5 | 0.07 | 4 | 0.05 | 3 |
| str-030 | Tr | 9 | -0.82 | 36.43 | 66 | 29.68 | 96.08 | 13.96 | 0.06 | 4 | 0.05 | 5 | 0.06 | 3 |
| str-031 | Tr | 9 | -0.82 | 38.38 | 69 | 32.14 | 87.44 | 23.32 | 0.08 | 3 | 0.04 | 4 | 0.05 | 6 |
| str-032 | Tr | 10 | -0.79 | 38.15 | 76 | 29.66 | 78.04 | 21.05 | 0.05 | 7 | 0.08 | 6 | 0.07 | 6 |
| str-033 | Tr | 10 | -0.79 | 37.44 | 80 | 29.57 | 76.32 | 21.09 | 0.05 | 5 | 0.05 | 6 | 0.06 | 9 |
| str-034 | Ts | 10 | -0.79 | 37.15 | 80 | 29.96 | 95.96 | 12.88 | 0.05 | 1 | 0.01 | 4 | 0.04 | 6 |
| str-035 | Tr | 11 | -0.76 | 35.70 | 59 | 29.73 | 78.06 | 20.88 | 0.03 | 2 | 0.03 | 3 | 0.04 | 9 |
| str-036 | Tr | 12 | -0.73 | 37.67 | 59 | 30.78 | 70.96 | 27.58 | 0.06 | 7 | 0.10 | 5 | 0.07 | 6 |
| str-037 | Ts | 12 | -0.73 | 37.67 | 65 | 30.78 | 72.18 | 21.05 | 0.08 | 4 | 0.05 | 5 | 0.06 | 6 |
| str-038 | Tr | 12 | -0.73 | 36.82 | 54 | 29.52 | 66.78 | 26.20 | 0.06 | 4 | 0.06 | 4 | 0.06 | 9 |
| str-039 | Ts | 12 | -0.73 | 36.73 | 65 | 29.61 | 92.24 | 19.65 | 0.08 | 4 | 0.05 | 5 | 0.06 | 6 |
| str-040 | Tr | 12 | -0.73 | 37.67 | 78 | 30.78 | 79.40 | 13.95 | 0.05 | 1 | 0.01 | 4 | 0.05 | 6 |
| str-041 | Tr | 12 | -0.73 | 36.16 | 65 | 29.96 | 80.28 | 19.34 | 0.08 | 4 | 0.05 | 5 | 0.06 | 6 |
| str-042 | Tr | 13 | -0.70 | 36.45 | 60 | 30.72 | 69.54 | 15.69 | 0.06 | 3 | 0.04 | 3 | 0.04 | 7 |
| str-043 | Tr | 13 | -0.70 | 34.06 | 60 | 29.26 | 81.08 | 15.80 | 0.06 | 3 | 0.04 | 3 | 0.04 | 6 |
| str-044 | Tr | 13 | -0.70 | 34.48 | 76 | 29.00 | 81.88 | 23.34 | 0.08 | 3 | 0.03 | 5 | 0.06 | 6 |
| str-045 | Tr | 13 | -0.70 | 39.31 | 48 | 31.95 | 92.78 | 18.38 | 0.09 | 5 | 0.08 | 3 | 0.05 | 9 |
| str-046 | Tr | 14 | -0.67 | 34.90 | 75 | 29.60 | 75.88 | 14.00 | 0.05 | 1 | 0.01 | 4 | 0.05 | 6 |
| str-047 | Tr | 14 | -0.67 | 36.31 | 59 | 29.68 | 79.14 | 19.17 | 0.06 | 4 | 0.05 | 5 | 0.07 | 9 |
| str-048 | Tr | 14 | -0.67 | 35.92 | 42 | 29.96 | 92.90 | 20.68 | 0.06 | 2 | 0.04 | 6 | 0.11 | 7 |
| str-049 | Tr | 15 | -0.64 | 36.52 | 44 | 29.80 | 83.64 | 25.18 | 0.09 | 2 | 0.03 | 4 | 0.07 | 9 |
| str-050 | Ts | 15 | -0.64 | 33.74 | 71 | 29.80 | 63.42 | 8.30 | 0.05 | 5 | 0.07 | 3 | 0.04 | 3 |
| str-051 | Tr | 15 | -0.64 | 36.36 | 76 | 30.72 | 76.18 | 16.41 | 0.05 | 4 | 0.05 | 6 | 0.07 | 6 |
| str-052 | Tr | 15 | -0.64 | 36.72 | 79 | 29.73 | 69.18 | 8.49 | 0.05 | 2 | 0.02 | 4 | 0.04 | 6 |
| str-053 | Tr | 16 | -0.62 | 35.33 | 54 | 29.50 | 69.98 | 21.67 | 0.06 | 6 | 0.09 | 4 | 0.06 | 6 |
| str-054 | Tr | 16 | -0.62 | 37.28 | 81 | 30.03 | 81.56 | 20.22 | 0.06 | 1 | 0.01 | 6 | 0.07 | 6 |
| str-055 | Tr | 17 | -0.59 | 38.28 | 73 | 30.78 | 76.70 | 20.46 | 0.08 | 4 | 0.05 | 5 | 0.06 | 6 |
| str-056 | Ts | 17 | -0.59 | 35.14 | 73 | 29.47 | 74.36 | 20.46 | 0.08 | 4 | 0.05 | 5 | 0.06 | 6 |
| str-057 | Tr | 17 | -0.59 | 38.28 | 27 | 30.78 | 79.64 | 9.67 | 0.05 | 2 | 0.06 | 2 | 0.06 | 3 |
| str-058 | Ts | 17 | -0.59 | 38.28 | 73 | 30.78 | 73.88 | 19.05 | 0.08 | 4 | 0.05 | 5 | 0.06 | 6 |
| str-059 | Tr | 17 | -0.59 | 26.75 | 57 | 29.61 | 37.74 | 18.39 | 0.08 | 5 | 0.07 | 3 | 0.04 | 9 |
| str-060 | Tr | 18 | -0.57 | 39.00 | 55 | 31.20 | 70.56 | 22.42 | 0.08 | 3 | 0.04 | 6 | 0.09 | 9 |
| str-061 | Tr | 18 | -0.57 | 38.44 | 66 | 31.56 | 69.48 | 13.62 | 0.03 | 3 | 0.04 | 3 | 0.04 | 9 |
| str-062 | Tr | 18 | -0.57 | 38.96 | 60 | 30.03 | 81.16 | 13.28 | 0.03 | 2 | 0.03 | 3 | 0.04 | 12 |
| str-063 | Tr | 18.4 | -0.56 | 32.91 | 48 | 30.19 | 58.68 | 11.26 | 0.03 | 0 | 0 | 6 | 0.11 | 2 |
| str-064 | Ts | 19 | -0.55 | 35.36 | 66 | 29.68 | 66.42 | 16.70 | 0.06 | 4 | 0.05 | 5 | 0.07 | 6 |
| str-065 | Tr | 19 | -0.55 | 33.10 | 57 | 28.99 | 64.94 | 13.63 | 0.05 | 1 | 0.02 | 3 | 0.05 | 5 |
| str-066 | Tr | 20 | -0.52 | 33.32 | 45 | 29.76 | 66.34 | 7.56 | 0.04 | 3 | 0.06 | 5 | 0.10 | 3 |
| str-067 | Tr | 20 | -0.52 | 36.94 | 45 | 30.49 | 66.54 | 7.56 | 0.04 | 3 | 0.06 | 5 | 0.10 | 3 |
| str-068 | Ts | 20 | -0.52 | 37.29 | 79 | 30.03 | 95.00 | 22.16 | 0.07 | 1 | 0.01 | 6 | 0.07 | 9 |
| str-069 | Tr | 20.3 | -0.52 | 32.94 | 48 | 29.53 | 63.34 | 19.92 | 0.09 | 4 | 0.07 | 3 | 0.05 | 9 |
| str-070 | Tr | 21 | -0.50 | 36.29 | 60 | 29.68 | 58.96 | 16.88 | 0.06 | 4 | 0.06 | 5 | 0.07 | 6 |
| str-071 | Ts | 23 | -0.46 | 34.33 | 81 | 29.69 | 70.66 | 14.82 | 0.05 | 1 | 0.01 | 4 | 0.04 | 6 |
| str-072 | Ts | 23 | -0.46 | 36.39 | 40 | 29.96 | 85.68 | 16.47 | 0.06 | 3 | 0.06 | 4 | 0.08 | 7 |
| str-073 | Tr | 24 | -0.44 | 32.23 | 53 | 29.76 | 67.92 | 12.93 | 0.06 | 3 | 0.05 | 5 | 0.09 | 0 |
| str-074 | Tr | 24 | -0.44 | 33.58 | 54 | 29.76 | 63.30 | 18.37 | 0.05 | 0 | 0 | 6 | 0.10 | 5 |
| str-075 | Ts | 25 | -0.42 | 36.76 | 78 | 29.80 | 99.58 | 13.92 | 0.05 | 1 | 0.01 | 4 | 0.05 | 6 |
| str-076 | Tr | 25 | -0.42 | 34.90 | 72 | 29.60 | 75.70 | 9.91 | 0.05 | 5 | 0.06 | 4 | 0.05 | 3 |
| str-077 | Tr | 25 | -0.42 | 33.58 | 42 | 29.76 | 55.22 | 21.20 | 0.06 | 2 | 0.04 | 6 | 0.11 | 7 |
| str-078 | Tr | 25 | -0.42 | 36.16 | 82 | 29.96 | 94.70 | 9.27 | 0.05 | 2 | 0.02 | 4 | 0.04 | 6 |
| str-079 | Tr | 25 | -0.42 | 39.16 | 58 | 30.03 | 83.00 | 21.99 | 0.08 | 3 | 0.04 | 6 | 0.08 | 9 |
| str-080 | Tr | 25 | -0.42 | 38.65 | 54 | 31.58 | 67.58 | 18.37 | 0.05 | 0 | 0 | 6 | 0.10 | 5 |
| str-081 | Ts | 26 | -0.40 | 36.46 | 65 | 29.20 | 84.16 | 25.81 | 0.06 | 5 | 0.06 | 5 | 0.06 | 7 |
| str-082 | Ts | 27 | -0.38 | 38.49 | 67 | 30.68 | 77.04 | 30.48 | 0.08 | 4 | 0.05 | 7 | 0.09 | 6 |
| str-083 | Tr | 27 | -0.38 | 34.34 | 67 | 29.47 | 47.58 | 31.48 | 0.08 | 4 | 0.05 | 7 | 0.09 | 6 |
| str-084 | Ts | 27 | -0.38 | 39.25 | 51 | 30.90 | 74.76 | 22.99 | 0.08 | 3 | 0.04 | 4 | 0.06 | 9 |
| str-085 | Tr | 27 | -0.38 | 38.49 | 51 | 30.68 | 84.58 | 14.68 | 0.05 | 5 | 0.08 | 3 | 0.05 | 6 |
| str-086 | Ts | 29 | -0.35 | 36.99 | 85 | 29.80 | 97.10 | 8.41 | 0.05 | 2 | 0.02 | 4 | 0.04 | 6 |
| str-087 | Tr | 29 | -0.35 | 28.72 | 36 | 29.26 | 41.42 | 12.87 | 0.04 | 4 | 0.10 | 1 | 0.02 | 4 |
| str-088 | Tr | 30 | -0.33 | 39.53 | 58 | 29.88 | 75.38 | 16.79 | 0.08 | 5 | 0.07 | 5 | 0.07 | 9 |
| str-089 | Tr | 30 | -0.33 | 36.07 | 56 | 29.61 | 87.30 | 19.91 | 0.10 | 5 | 0.07 | 4 | 0.06 | 6 |
| str-090 | Tr | 30 | -0.33 | 37.46 | 65 | 31.58 | 76.16 | 11.95 | 0.05 | 4 | 0.05 | 4 | 0.05 | 6 |
| str-091 | Tr | 30.7 | -0.31 | 33.84 | 50 | 29.90 | 64.30 | 11.29 | 0.03 | 2 | 0.04 | 6 | 0.11 | 2 |
| str-092 | Ts | 32.5 | -0.28 | 38.91 | 55 | 29.92 | 68.68 | 16.62 | 0.08 | 4 | 0.06 | 4 | 0.06 | 9 |
| str-093 | Tr | 33 | -0.27 | 32.46 | 59 | 28.72 | 61.30 | 6.85 | 0.03 | 3 | 0.05 | 2 | 0.03 | 6 |
| str-094 | Tr | 33 | -0.27 | 35.72 | 69 | 30.66 | 64.08 | 10.19 | 0.03 | 1 | 0.01 | 3 | 0.04 | 3 |
| str-095 | Tr | 33 | -0.27 | 30.81 | 42 | 29.96 | 56.00 | 6.00 | 0.04 | 1 | 0.02 | 3 | 0.06 | 4 |
| str-096 | Tr | 33 | -0.27 | 34.48 | 61 | 29.00 | 76.96 | 13.33 | 0.05 | 5 | 0.08 | 4 | 0.06 | 3 |
| str-097 | Tr | 33 | -0.27 | 32.36 | 60 | 29.96 | 60.52 | 15.80 | 0.06 | 3 | 0.04 | 3 | 0.04 | 6 |
| str-098 | Tr | 33 | -0.27 | 34.40 | 68 | 28.72 | 63.74 | 10.47 | 0.03 | 1 | 0.01 | 4 | 0.05 | 3 |
| str-099 | Tr | 33 | -0.27 | 32.81 | 49 | 29.50 | 46.08 | 11.81 | 0.06 | 1 | 0.02 | 4 | 0.07 | 6 |
| str-100 | Tr | 33 | -0.27 | 34.53 | 47 | 28.91 | 66.94 | 9.70 | 0.05 | 1 | 0.02 | 4 | 0.07 | 4 |
| str-101 | Ts | 33.1 | -0.27 | 30.81 | 48 | 29.96 | 55.66 | 8.86 | 0.05 | 1 | 0.02 | 4 | 0.07 | 2 |
| str-102 | Tr | 34 | -0.26 | 33.97 | 56 | 29.38 | 69.58 | 21.24 | 0.06 | 2 | 0.03 | 4 | 0.06 | 5 |
| str-103 | Tr | 34 | -0.26 | 32.32 | 65 | 28.82 | 53.44 | 6.68 | 0.04 | 3 | 0.04 | 3 | 0.04 | 9 |
| str-104 | Tr | 34 | -0.26 | 36.14 | 40 | 29.61 | 85.36 | 16.47 | 0.06 | 3 | 0.06 | 4 | 0.08 | 7 |
| str-105 | Tr | 34 | -0.26 | 34.88 | 33 | 28.92 | 54.82 | 24.83 | 0.06 | 4 | 0.10 | 3 | 0.07 | 3 |
| str-106 | Ts | 35 | -0.24 | 36.98 | 85 | 29.80 | 95.56 | 16.80 | 0.05 | 2 | 0.02 | 4 | 0.04 | 6 |
| str-107 | Ts | 36 | -0.22 | 36.39 | 55 | 29.96 | 92.92 | 19.06 | 0.06 | 3 | 0.04 | 5 | 0.07 | 9 |
| str-108 | Tr | 36 | -0.22 | 33.37 | 36 | 29.42 | 70.68 | 31.99 | 0.06 | 4 | 0.09 | 5 | 0.11 | 3 |
| str-109 | Ts | 36 | -0.22 | 38.92 | 51 | 31.24 | 75.48 | 22.25 | 0.08 | 3 | 0.04 | 4 | 0.06 | 9 |
| str-110 | Tr | 36 | -0.22 | 36.37 | 38 | 29.76 | 70.66 | 7.18 | 0.06 | 4 | 0.07 | 2 | 0.04 | 9 |
| str-111 | Tr | 36 | -0.22 | 32.40 | 81 | 29.68 | 55.20 | 11.82 | 0.05 | 1 | 0.01 | 4 | 0.04 | 6 |
| str-112 | Ts | 36 | -0.22 | 38.92 | 51 | 31.24 | 69.00 | 22.59 | 0.08 | 3 | 0.04 | 4 | 0.06 | 9 |
| str-113 | Tr | 37 | -0.21 | 38.50 | 73 | 29.68 | 77.60 | 21.20 | 0.05 | 4 | 0.05 | 5 | 0.06 | 7 |
| str-114 | Tr | 37 | -0.21 | 35.56 | 71 | 28.38 | 62.82 | 5.80 | 0.10 | 2 | 0.03 | 2 | 0.03 | 6 |
| str-115 | Tr | 37 | -0.21 | 37.06 | 76 | 30.03 | 86.00 | 23.80 | 0.07 | 1 | 0.01 | 6 | 0.07 | 9 |
| str-116 | Ts | 37 | -0.21 | 36.18 | 73 | 29.73 | 83.00 | 23.46 | 0.06 | 4 | 0.05 | 6 | 0.07 | 6 |
| str-117 | Tr | 37.3 | -0.20 | 41.72 | 68 | 30.20 | 78.72 | 12.70 | 0.03 | 2 | 0.02 | 3 | 0.04 | 9 |
| str-118 | Tr | 38 | -0.19 | 36.85 | 54 | 29.22 | 92.44 | 22.74 | 0.08 | 3 | 0.04 | 6 | 0.09 | 9 |
| str-119 | Tr | 38 | -0.19 | 39.45 | 55 | 30.90 | 80.60 | 22.32 | 0.08 | 3 | 0.04 | 4 | 0.06 | 9 |
| str-120 | Tr | 38 | -0.19 | 38.63 | 68 | 30.27 | 71.56 | 31.29 | 0.07 | 3 | 0.04 | 7 | 0.09 | 6 |
| str-121 | Ts | 38.3 | -0.19 | 37.81 | 65 | 30.85 | 83.84 | 18.44 | 0.08 | 4 | 0.05 | 4 | 0.05 | 6 |
| str-122 | Tr | 38.6 | -0.18 | 38.37 | 51 | 30.17 | 81.46 | 17.95 | 0.08 | 4 | 0.06 | 4 | 0.06 | 9 |
| str-123 | Ts | 39.8 | -0.16 | 31.17 | 42 | 29.96 | 47.64 | 6.22 | 0.04 | 1 | 0.02 | 4 | 0.08 | 3 |
| str-124 | Tr | 40 | -0.16 | 35.67 | 49 | 28.42 | 65.52 | 16.94 | 0.07 | 3 | 0.05 | 4 | 0.07 | 6 |
| str-125 | Tr | 40 | -0.16 | 31.84 | 47 | 29.61 | 52.36 | 19.98 | 0.06 | 4 | 0.07 | 4 | 0.07 | 3 |
| str-126 | Ts | 40.5 | -0.15 | 38.58 | 54 | 30.17 | 76.98 | 18.31 | 0.08 | 4 | 0.06 | 4 | 0.06 | 9 |
| str-127 | Tr | 41 | -0.14 | 37.35 | 27 | 30.93 | 80.96 | 8.64 | 0.09 | 2 | 0.07 | 1 | 0.03 | 0 |
| str-128 | Ts | 41 | -0.14 | 38.27 | 68 | 30.68 | 69.62 | 31.90 | 0.08 | 4 | 0.05 | 7 | 0.09 | 6 |
| str-129 | Tr | 41 | -0.14 | 25.97 | 44 | 29.42 | 29.90 | 13.38 | 0.09 | 4 | 0.07 | 3 | 0.05 | 6 |
| str-130 | Ts | 42 | -0.13 | 37.17 | 75 | 30.66 | 81.84 | 11.53 | 0.05 | 5 | 0.06 | 4 | 0.05 | 3 |
| str-131 | Tr | 42.9 | -0.11 | 31.67 | 41 | 29.49 | 46.78 | 7.58 | 0.04 | 1 | 0.02 | 4 | 0.08 | 3 |
| str-132 | Tr | 43 | -0.11 | 34.46 | 37 | 29.61 | 81.70 | 24.25 | 0.06 | 5 | 0.11 | 3 | 0.07 | 3 |
| str-133 | Tr | 43 | -0.11 | 31.68 | 76 | 29.47 | 56.96 | 2.89 | 0.10 | 2 | 0.02 | 2 | 0.02 | 6 |
| str-134 | Tr | 44 | -0.09 | 35.78 | 68 | 29.55 | 55.08 | 10.48 | 0.05 | 5 | 0.07 | 4 | 0.05 | 6 |
| str-135 | Tr | 44 | -0.09 | 37.84 | 77 | 30.35 | 75.24 | 10.00 | 0.10 | 2 | 0.02 | 4 | 0.05 | 6 |
| str-136 | Tr | 44 | -0.09 | 34.83 | 61 | 29.68 | 66.66 | 15.97 | 0.04 | 5 | 0.07 | 4 | 0.06 | 6 |
| str-137 | Tr | 44 | -0.09 | 35.91 | 61 | 29.43 | 78.64 | 21.70 | 0.06 | 4 | 0.06 | 5 | 0.07 | 6 |
| str-138 | Ts | 46 | -0.06 | 36.01 | 64 | 30.66 | 69.56 | 12.48 | 0.05 | 5 | 0.07 | 4 | 0.06 | 3 |
| str-139 | Tr | 47 | -0.05 | 35.86 | 57 | 30.85 | 79.52 | 23.60 | 0.06 | 4 | 0.06 | 5 | 0.07 | 9 |
| str-140 | Tr | 47 | -0.05 | 37.35 | 55 | 30.93 | 84.76 | 16.49 | 0.09 | 4 | 0.06 | 4 | 0.06 | 6 |
| str-141 | Ts | 47 | -0.05 | 35.86 | 55 | 30.85 | 68.74 | 16.49 | 0.09 | 4 | 0.06 | 4 | 0.06 | 6 |
| str-142 | Ts | 47 | -0.05 | 37.12 | 44 | 30.78 | 87.90 | 13.38 | 0.09 | 4 | 0.07 | 3 | 0.05 | 6 |
| str-143 | Ts | 48 | -0.03 | 33.25 | 42 | 29.42 | 51.34 | 1.10 | 0.05 | 4 | 0.08 | 2 | 0.04 | 6 |
| str-144 | Ts | 49 | -0.02 | 33.92 | 59 | 29.34 | 77.06 | 13.87 | 0.04 | 5 | 0.07 | 3 | 0.04 | 6 |
| str-145 | Tr | 49 | -0.02 | 35.41 | 53 | 29.47 | 51.68 | 28.36 | 0.08 | 4 | 0.06 | 4 | 0.06 | 7 |
| str-146 | Tr | 50 | 0.00 | 32.63 | 41 | 29.86 | 54.26 | 10.72 | 0.06 | 2 | 0.04 | 5 | 0.10 | 4 |
| str-147 | Tr | 50 | 0.00 | 34.56 | 68 | 29.69 | 92.56 | 17.03 | 0.06 | 3 | 0.04 | 4 | 0.05 | 7 |
| str-148 | Ts | 50 | 0.00 | 36.10 | 51 | 29.38 | 79.20 | 25.16 | 0.06 | 3 | 0.05 | 5 | 0.08 | 6 |
| str-149 | Tr | 50 | 0.00 | 33.62 | 90 | 29.76 | 53.10 | 13.39 | 0.06 | 3 | 0.03 | 4 | 0.04 | 3 |
| str-150 | Ts | 51 | 0.02 | 36.74 | 66 | 31.20 | 77.30 | 12.45 | 0.03 | 3 | 0.04 | 3 | 0.04 | 10 |
| str-151 | Tr | 51 | 0.02 | 36.74 | 34 | 31.20 | 64.06 | 19.38 | 0.06 | 4 | 0.09 | 2 | 0.05 | 3 |
| str-152 | Tr | 51 | 0.02 | 39.00 | 40 | 31.20 | 79.26 | 13.16 | 0.08 | 3 | 0.06 | 3 | 0.06 | 6 |
| str-153 | Ts | 51 | 0.02 | 36.60 | 40 | 30.52 | 73.62 | 13.16 | 0.08 | 3 | 0.06 | 3 | 0.06 | 6 |
| str-154 | Tr | 51 | 0.02 | 32.21 | 65 | 28.66 | 48.90 | 16.32 | 0.05 | 6 | 0.08 | 4 | 0.06 | 3 |
| str-155 | Ts | 53 | 0.05 | 39.78 | 56 | 30.75 | 75.82 | 22.64 | 0.08 | 4 | 0.06 | 4 | 0.06 | 9 |
| str-156 | Tr | 53 | 0.05 | 33.70 | 53 | 29.23 | 61.82 | 9.99 | 0.06 | 4 | 0.07 | 3 | 0.05 | 6 |
| str-157 | Tr | 53.1 | 0.05 | 33.05 | 46 | 30.03 | 51.54 | 16.62 | 0.05 | 1 | 0.02 | 6 | 0.12 | 2 |
| str-158 | Tr | 54 | 0.06 | 40.00 | 57 | 31.12 | 85.90 | 17.55 | 0.05 | 3 | 0.04 | 3 | 0.04 | 6 |
| str-159 | Tr | 54 | 0.06 | 31.45 | 75 | 29.67 | 46.74 | 10.28 | 0.05 | 7 | 0.09 | 5 | 0.06 | 3 |
| str-160 | Ts | 54 | 0.06 | 33.59 | 57 | 29.61 | 52.62 | 17.55 | 0.05 | 3 | 0.04 | 3 | 0.04 | 6 |
| str-161 | Tr | 54 | 0.06 | 33.59 | 53 | 29.61 | 63.32 | 17.00 | 0.08 | 4 | 0.06 | 5 | 0.07 | 9 |
| str-162 | Tr | 54 | 0.06 | 38.67 | 26 | 30.18 | 75.30 | 22.81 | 0.04 | 1 | 0.03 | 6 | 0.17 | 3 |
| str-163 | Tr | 59 | 0.14 | 35.94 | 44 | 30.62 | 60.00 | 11.55 | 0.06 | 2 | 0.04 | 5 | 0.10 | 3 |
| str-164 | Tr | 62 | 0.19 | 37.24 | 59 | 30.78 | 68.24 | 17.40 | 0.08 | 4 | 0.06 | 5 | 0.07 | 6 |
| str-165 | Tr | 63 | 0.21 | 37.20 | 80 | 30.00 | 95.66 | 17.39 | 0.05 | 1 | 0.01 | 5 | 0.05 | 9 |
| str-166 | Tr | 64 | 0.22 | 37.94 | 72 | 30.39 | 71.90 | 17.60 | 0.06 | 4 | 0.05 | 5 | 0.06 | 9 |
| str-167 | Tr | 64 | 0.22 | 37.13 | 68 | 29.68 | 75.10 | 15.51 | 0.05 | 7 | 0.09 | 4 | 0.05 | 3 |
| str-168 | Tr | 65 | 0.24 | 37.65 | 69 | 30.59 | 76.86 | 6.25 | 0.04 | 2 | 0.03 | 4 | 0.05 | 3 |
| str-169 | Tr | 65 | 0.24 | 34.93 | 67 | 28.92 | 63.52 | 18.04 | 0.05 | 5 | 0.07 | 6 | 0.08 | 3 |
| str-170 | Ts | 66 | 0.26 | 38.75 | 76 | 29.68 | 82.82 | 19.70 | 0.05 | 4 | 0.05 | 5 | 0.06 | 7 |
| str-171 | Tr | 66 | 0.26 | 34.07 | 42 | 29.93 | 48.02 | 13.21 | 0.10 | 3 | 0.06 | 1 | 0.02 | 6 |
| str-172 | Tr | 68 | 0.29 | 31.26 | 35 | 29.36 | 51.52 | 18.99 | 0.06 | 5 | 0.11 | 2 | 0.05 | 3 |
| str-173 | Tr | 69 | 0.31 | 34.96 | 42 | 29.77 | 74.96 | 32.04 | 0.06 | 1 | 0.02 | 8 | 0.15 | 7 |
| str-174 | Tr | 69 | 0.31 | 37.79 | 59 | 30.55 | 54.64 | 7.39 | 0.04 | 6 | 0.09 | 3 | 0.04 | 6 |
| str-175 | Tr | 70 | 0.33 | 36.23 | 37 | 31.72 | 58.34 | 16.21 | 0.08 | 5 | 0.10 | 3 | 0.06 | 3 |
| str-176 | Tr | 71 | 0.35 | 31.41 | 54 | 29.73 | 52.58 | 8.29 | 0.05 | 2 | 0.03 | 3 | 0.05 | 3 |
| str-177 | Tr | 72 | 0.36 | 36.48 | 70 | 30.66 | 72.40 | 11.37 | 0.05 | 5 | 0.07 | 4 | 0.05 | 3 |
| str-178 | Tr | 72 | 0.36 | 34.35 | 46 | 29.26 | 79.02 | 8.49 | 0.10 | 4 | 0.06 | 1 | 0.02 | 10 |
| str-179 | Tr | 73 | 0.38 | 35.81 | 47 | 30.83 | 85.44 | 32.20 | 0.10 | 3 | 0.05 | 7 | 0.12 | 7 |
| str-180 | Tr | 75.1 | 0.42 | 35.90 | 54 | 30.93 | 70.82 | 9.61 | 0.05 | 4 | 0.06 | 3 | 0.05 | 6 |
| str-181 | Tr | 76 | 0.44 | 35.80 | 60 | 30.55 | 69.42 | 9.95 | 0.05 | 6 | 0.09 | 3 | 0.05 | 3 |
| str-182 | Ts | 77 | 0.46 | 28.52 | 37 | 29.73 | 41.26 | 9.86 | 0.06 | 2 | 0.05 | 3 | 0.07 | 3 |
| str-183 | Tr | 77 | 0.46 | 37.07 | 65 | 28.87 | 78.80 | 23.43 | 0.06 | 4 | 0.05 | 6 | 0.08 | 3 |
| str-184 | Tr | 78 | 0.48 | 33.30 | 49 | 28.77 | 45.92 | 16.65 | 0.06 | 3 | 0.05 | 4 | 0.07 | 4 |
| str-185 | Tr | 82 | 0.57 | 39.25 | 71 | 31.19 | 69.50 | 11.60 | 0.05 | 7 | 0.09 | 4 | 0.05 | 3 |
| str-186 | Tr | 84.2 | 0.62 | 32.85 | 50 | 29.36 | 52.68 | 4.97 | 0.05 | 5 | 0.09 | 2 | 0.03 | 6 |
| str-187 | Tr | 85 | 0.64 | 30.10 | 40 | 29.73 | 48.06 | 9.52 | 0.06 | 2 | 0.04 | 3 | 0.06 | 3 |
| str-188 | Tr | 85 | 0.64 | 29.60 | 39 | 29.90 | 46.72 | 8.22 | 0.05 | 1 | 0.02 | 3 | 0.07 | 6 |
| str-189 | Tr | 86 | 0.67 | 38.41 | 71 | 30.66 | 86.94 | 9.08 | 0.07 | 5 | 0.06 | 4 | 0.05 | 6 |
| str-190 | Ts | 87 | 0.70 | 35.64 | 62 | 30.72 | 64.42 | 8.08 | 0.05 | 5 | 0.07 | 3 | 0.04 | 3 |
| str-191 | Tr | 88.5 | 0.74 | 42.16 | 88 | 31.19 | 110.46 | 12.11 | 0.06 | 4 | 0.04 | 4 | 0.04 | 9 |
| str-192 | Tr | 89 | 0.76 | 33.22 | 56 | 29.20 | 69.82 | 21.74 | 0.04 | 1 | 0.02 | 7 | 0.11 | 6 |
| str-193 | Tr | 89 | 0.76 | 35.32 | 36 | 30.05 | 66.28 | 11.61 | 0.10 | 6 | 0.12 | 1 | 0.02 | 9 |
| str-194 | Tr | 90 | 0.79 | 38.85 | 33 | 31.19 | 73.66 | 3.02 | 0.04 | 2 | 0.05 | 3 | 0.07 | 6 |
| str-195 | Tr | 90 | 0.79 | 34.78 | 65 | 31.56 | 55.86 | 11.04 | 0.05 | 7 | 0.10 | 4 | 0.06 | 3 |
| str-196 | Tr | 92 | 0.86 | 35.82 | 67 | 29.69 | 66.02 | 5.37 | 0.04 | 3 | 0.04 | 4 | 0.05 | 3 |
| str-197 | Ts | 94 | 0.94 | 36.24 | 67 | 30.66 | 69.22 | 10.99 | 0.05 | 5 | 0.07 | 4 | 0.06 | 3 |
| str-198 | Tr | 96 | 1.03 | 37.15 | 72 | 29.50 | 71.06 | 5.79 | 0.04 | 6 | 0.07 | 4 | 0.05 | 5 |
| str-199 | Tr | 97 | 1.08 | 36.84 | 40 | 30.62 | 77.66 | 8.64 | 0.07 | 5 | 0.09 | 5 | 0.09 | 6 |
| str-200 | Tr | 98 | 1.14 | 30.77 | 50 | 28.79 | 54.02 | 13.81 | 0.09 | 2 | 0.04 | 2 | 0.04 | 3 |
| str-201 | Tr | 100 | 1.28 | 34.34 | 61 | 29.93 | 45.04 | 9.64 | 0.04 | 6 | 0.08 | 4 | 0.06 | 6 |
| str-202 | Tr | 100 | 1.28 | 35.54 | 44 | 29.47 | 75.42 | 12.19 | 0.10 | 3 | 0.06 | 1 | 0.02 | 6 |
| str-203 | Tr | 100 | 1.28 | 38.11 | 51 | 30.52 | 50.22 | 11.44 | 0.07 | 3 | 0.05 | 5 | 0.08 | 9 |
| str-204 | Ts | 100 | 1.28 | 36.12 | 68 | 30.72 | 71.34 | 9.96 | 0.05 | 5 | 0.07 | 3 | 0.04 | 3 |
| str-205 | Tr | 100 | 1.28 | 37.66 | 58 | 29.80 | 67.12 | 19.70 | 0.06 | 3 | 0.04 | 6 | 0.08 | 6 |
| str-206 | Tr | 10 | -0.79 | 35.90 | 75 | 29.61 | 87.72 | 22.09 | 0.08 | 4 | 0.05 | 4 | 0.05 | 12 |
| str-207 | Tr | 11 | -0.76 | 37.40 | 44 | 31.83 | 69.46 | 21.90 | 0.08 | 4 | 0.07 | 4 | 0.07 | 6 |
| str-208 | Tr | 13 | -0.70 | 34.48 | 57 | 29.00 | 85.44 | 13.26 | 0.06 | 3 | 0.04 | 3 | 0.04 | 12 |
| str-209 | Tr | 20 | -0.52 | 35.67 | 72 | 29.43 | 75.18 | 6.07 | 0.04 | 2 | 0.02 | 4 | 0.05 | 12 |
| str-210 | Tr | 25 | -0.42 | 35.80 | 68 | 29.14 | 94.00 | 18.43 | 0.05 | 3 | 0.04 | 6 | 0.08 | 6 |
| str-211 | Ts | 37 | -0.21 | 34.78 | 56 | 29.00 | 80.46 | 14.29 | 0.06 | 3 | 0.04 | 3 | 0.04 | 12 |
| str-212 | Tr | 61 | 0.17 | 36.12 | 71 | 29.34 | 92.02 | 14.94 | 0.06 | 4 | 0.05 | 4 | 0.05 | 12 |
| str-213 | Ts | 95 | 0.98 | 34.90 | 60 | 29.53 | 88.50 | 13.61 | 0.06 | 4 | 0.06 | 3 | 0.04 | 12 |
| str-214 | Tr | 78 | 0.48 | 32.56 | 60 | 29.61 | 51.58 | 21.69 | 0.10 | 4 | 0.06 | 4 | 0.06 | 0 |
| str-215 | Tr | 100 | 1.28 | 33.84 | 46 | 29.32 | 66.58 | 11.97 | 0.06 | 6 | 0.10 | 2 | 0.03 | 12 |
|  |  |  |  |  |  |  |  |  |  |  |  |  |  |  |
| str-216 | Tr | 14 | -0.67 | 34.90 | 75 | 29.60 | 75.88 | 14.00 | 0.05 | 1 | 0.01 | 4 | 0.05 | 6 |
| str-217 | Tr | 14 | -0.67 | 36.31 | 59 | 29.68 | 79.14 | 19.17 | 0.06 | 4 | 0.05 | 5 | 0.07 | 9 |

Table-2 Represents supplementary data for A Total of 11 descriptors were generated for each of the 217 compounds using Codessa 2.1 tool

| Bioavailability | G INDEX | Number of single bonds | SAAA | YZ Shadow | HASA2[Zefirov's_PC] | Max partial charge for a H atom [Zefirov's_PC] | Number of O atoms | Relativenumberof O Atoms | Number of N atoms | Relative number of N atoms | Number of aromatic bonds |
| --- | --- | --- | --- | --- | --- | --- | --- | --- | --- | --- | --- |
| 0 | 29.7476 | 38 | 29.9592 | 39.9805 | 11.1941 | 0.1019 | 1 | 0.0222 | 4 | 0.0889 | 3 |
| 1 | 35.4908 | 74 | 29.6763 | 71.6413 | 14.1248 | 0.0597 | 4 | 0.0465 | 5 | 0.0581 | 6 |
| 1.6 | 33.5516 | 53 | 29.3772 | 67.8411 | 24.2433 | 0.0839 | 3 | 0.0462 | 4 | 0.0615 | 6 |
| 2 | 34.3298 | 40 | 29.6947 | 77.9814 | 15.7244 | 0.0604 | 3 | 0.0566 | 4 | 0.0755 | 6 |
| 2 | 33.0241 | 44 | 29.5477 | 58.441 | 23.5789 | 0.0562 | 5 | 0.0962 | 4 | 0.0769 | 3 |
| 3 | 37.1635 | 77 | 29.0459 | 94.5617 | 15.3234 | 0.0586 | 4 | 0.046 | 5 | 0.0575 | 6 |
| 3.4 | 33.9768 | 54 | 29.339 | 69.8212 | 26.1077 | 0.0995 | 4 | 0.0606 | 4 | 0.0606 | 6 |
| 4 | 36.3443 | 61 | 29.2785 | 80.5814 | 24.7135 | 0.061 | 5 | 0.0685 | 6 | 0.0822 | 7 |
| 4 | 37.0397 | 86 | 29.7278 | 81.5215 | 11.9513 | 0.0485 | 1 | 0.0103 | 4 | 0.0412 | 6 |
| 4 | 37.6003 | 61 | 29.9592 | 97.2418 | 19.3977 | 0.0599 | 4 | 0.0548 | 6 | 0.0822 | 3 |
| 5 | 37.4891 | 79 | 29.6947 | 75.5813 | 15.2451 | 0.0586 | 4 | 0.0444 | 5 | 0.0556 | 6 |
| 5 | 36.2973 | 81 | 29.6763 | 82.8415 | 18.9261 | 0.0599 | 2 | 0.0215 | 5 | 0.0538 | 9 |
| 5 | 37.4991 | 67 | 29.86 | 97.3218 | 12.4968 | 0.0363 | 3 | 0.039 | 4 | 0.0519 | 7 |
| 5.1 | 35.4204 | 66 | 29.7567 | 75.1613 | 19.074 | 0.0546 | 3 | 0.0395 | 5 | 0.0658 | 6 |
| 5.1 | 35.4204 | 66 | 29.7567 | 70.5412 | 18.8082 | 0.0546 | 3 | 0.0395 | 5 | 0.0658 | 6 |
| 5.7 | 37.6345 | 68 | 30.7802 | 84.3615 | 18.8789 | 0.0842 | 4 | 0.0506 | 5 | 0.0633 | 6 |
| 6 | 39.7068 | 81 | 30.6214 | 98.5419 | 12.1375 | 0.0727 | 5 | 0.0543 | 5 | 0.0543 | 5 |
| 7 | 32.5052 | 68 | 29.9014 | 56.9609 | 20.1838 | 0.0842 | 4 | 0.0506 | 5 | 0.0633 | 6 |
| 7 | 37.6345 | 84 | 30.7802 | 71.1612 | 13.2336 | 0.0485 | 1 | 0.0106 | 4 | 0.0426 | 6 |
| 7 | 37.6345 | 68 | 30.7802 | 74.8213 | 20.368 | 0.0842 | 4 | 0.0506 | 5 | 0.0633 | 6 |
| 7 | 36.6277 | 37 | 29.9592 | 87.9016 | 13.8261 | 0.054 | 1 | 0.0204 | 3 | 0.0612 | 7 |
| 7 | 37.6673 | 65 | 30.7802 | 80.2014 | 20.6192 | 0.0842 | 4 | 0.0519 | 5 | 0.0649 | 6 |
| 7.3 | 34.8413 | 49 | 30.0254 | 72.6413 | 13.4255 | 0.0342 | 1 | 0.0169 | 6 | 0.1017 | 5 |
| 7.9 | 36.4691 | 78 | 29.5219 | 86.9016 | 14.9144 | 0.0976 | 2 | 0.023 | 5 | 0.0575 | 6 |
| 8 | 37.0445 | 56 | 28.9926 | 81.7815 | 28.0367 | 0.0608 | 7 | 0.1045 | 5 | 0.0746 | 6 |
| 8 | 36.5471 | 62 | 29.5156 | 73.2213 | 20.7527 | 0.0309 | 2 | 0.0263 | 3 | 0.0395 | 9 |
| 8 | 35.2268 | 77 | 29.7278 | 80.4014 | 5.0325 | 0.0373 | 2 | 0.0222 | 3 | 0.0333 | 8 |
| 8.5 | 35.917 | 75 | 29.9592 | 95.4218 | 13.1939 | 0.0485 | 1 | 0.0118 | 4 | 0.0471 | 6 |
| 9 | 36.4796 | 70 | 30.6645 | 66.6211 | 11.7741 | 0.0545 | 5 | 0.0667 | 4 | 0.0533 | 3 |
| 9 | 36.4259 | 66 | 29.6763 | 96.0818 | 13.9581 | 0.0599 | 4 | 0.0519 | 5 | 0.0649 | 3 |
| 9 | 38.3794 | 69 | 32.1437 | 87.4416 | 23.3154 | 0.0842 | 3 | 0.037 | 4 | 0.0494 | 6 |
| 10 | 38.1507 | 76 | 29.6646 | 78.0414 | 21.0529 | 0.0546 | 7 | 0.0795 | 6 | 0.0682 | 6 |
| 10 | 37.4416 | 80 | 29.5675 | 76.3213 | 21.0894 | 0.0546 | 5 | 0.0526 | 6 | 0.0632 | 9 |
| 10 | 37.1541 | 80 | 29.9592 | 95.9618 | 12.8756 | 0.0485 | 1 | 0.011 | 4 | 0.044 | 6 |
| 11 | 35.7012 | 59 | 29.7278 | 78.0614 | 20.8785 | 0.0309 | 2 | 0.0274 | 3 | 0.0411 | 9 |
| 12 | 37.6673 | 59 | 30.7802 | 70.9612 | 27.5849 | 0.0608 | 7 | 0.1 | 5 | 0.0714 | 6 |
| 12 | 37.6673 | 65 | 30.7802 | 72.1813 | 21.0529 | 0.0842 | 4 | 0.0519 | 5 | 0.0649 | 6 |
| 12 | 36.8174 | 54 | 29.5156 | 66.7811 | 26.1978 | 0.06 | 4 | 0.0563 | 4 | 0.0563 | 9 |
| 12 | 36.7258 | 65 | 29.612 | 92.2417 | 19.6547 | 0.0842 | 4 | 0.0519 | 5 | 0.0649 | 6 |
| 12 | 37.6673 | 78 | 30.7802 | 79.4014 | 13.9452 | 0.0485 | 1 | 0.0114 | 4 | 0.0455 | 6 |
| 12 | 36.157 | 65 | 29.9592 | 80.2814 | 19.336 | 0.0842 | 4 | 0.0519 | 5 | 0.0649 | 6 |
| 13 | 36.445 | 60 | 30.7171 | 69.5412 | 15.6886 | 0.0579 | 3 | 0.0435 | 3 | 0.0435 | 7 |
| 13 | 34.0563 | 60 | 29.261 | 81.0815 | 15.7983 | 0.058 | 3 | 0.0435 | 3 | 0.0435 | 6 |
| 13 | 34.4766 | 76 | 28.9977 | 81.8815 | 23.3445 | 0.0842 | 3 | 0.0345 | 5 | 0.0575 | 6 |
| 13 | 39.3091 | 48 | 31.9484 | 92.7817 | 18.3788 | 0.0862 | 5 | 0.0806 | 3 | 0.0484 | 9 |
| 14 | 34.9043 | 75 | 29.5962 | 75.8813 | 13.9984 | 0.0485 | 1 | 0.0118 | 4 | 0.0471 | 6 |
| 14 | 36.3143 | 59 | 29.6763 | 79.1414 | 19.1659 | 0.0559 | 4 | 0.0541 | 5 | 0.0676 | 9 |
| 14 | 35.917 | 42 | 29.9592 | 92.9017 | 20.6811 | 0.0604 | 2 | 0.0364 | 6 | 0.1091 | 7 |
| 15 | 36.5241 | 44 | 29.8049 | 83.6415 | 25.177 | 0.0873 | 2 | 0.0333 | 4 | 0.0667 | 9 |
| 15 | 33.7379 | 71 | 29.8049 | 63.4211 | 8.3017 | 0.0545 | 5 | 0.0658 | 3 | 0.0395 | 3 |
| 15 | 36.3619 | 76 | 30.7171 | 76.1813 | 16.4105 | 0.053 | 4 | 0.0476 | 6 | 0.0714 | 6 |
| 15 | 36.7186 | 79 | 29.7278 | 69.1812 | 8.4855 | 0.0485 | 2 | 0.0225 | 4 | 0.0449 | 6 |
| 16 | 35.3328 | 54 | 29.4963 | 69.9812 | 21.6683 | 0.0561 | 6 | 0.0923 | 4 | 0.0615 | 6 |
| 16 | 37.2764 | 81 | 30.0254 | 81.5615 | 20.2223 | 0.0552 | 1 | 0.011 | 6 | 0.0659 | 6 |
| 17 | 38.2834 | 73 | 30.7802 | 76.7013 | 20.4592 | 0.0842 | 4 | 0.0482 | 5 | 0.0602 | 6 |
| 17 | 35.1361 | 73 | 29.4674 | 74.3613 | 20.4592 | 0.0842 | 4 | 0.0482 | 5 | 0.0602 | 6 |
| 17 | 38.2834 | 27 | 30.7802 | 79.6414 | 9.668 | 0.0528 | 2 | 0.0606 | 2 | 0.0606 | 3 |
| 17 | 38.2834 | 73 | 30.7802 | 73.8813 | 19.0483 | 0.0842 | 4 | 0.0482 | 5 | 0.0602 | 6 |
| 17 | 26.7464 | 57 | 29.612 | 37.7405 | 18.3865 | 0.0829 | 5 | 0.0704 | 3 | 0.0423 | 9 |
| 18 | 38.9963 | 55 | 31.1982 | 70.5612 | 22.4223 | 0.0842 | 3 | 0.0429 | 6 | 0.0857 | 9 |
| 18 | 38.4421 | 66 | 31.559 | 69.4812 | 13.6231 | 0.0317 | 3 | 0.0366 | 3 | 0.0366 | 9 |
| 18 | 38.9597 | 60 | 30.0326 | 81.1615 | 13.2763 | 0.0311 | 2 | 0.0256 | 3 | 0.0385 | 12 |
| 18.4 | 32.9096 | 48 | 30.1907 | 58.681 | 11.2633 | 0.0278 | 0 | 0 | 6 | 0.1111 | 2 |
| 19 | 35.3586 | 66 | 29.6763 | 66.4211 | 16.6973 | 0.0569 | 4 | 0.0533 | 5 | 0.0667 | 6 |
| 19 | 33.0961 | 57 | 28.9858 | 64.9411 | 13.6312 | 0.0533 | 1 | 0.0152 | 3 | 0.0455 | 5 |
| 20 | 33.316 | 45 | 29.7567 | 66.3411 | 7.5575 | 0.0351 | 3 | 0.0588 | 5 | 0.098 | 3 |
| 20 | 36.9442 | 45 | 30.4891 | 66.5411 | 7.5575 | 0.0351 | 3 | 0.0588 | 5 | 0.098 | 3 |
| 20 | 37.2898 | 79 | 30.0254 | 95.0018 | 22.1581 | 0.065 | 1 | 0.011 | 6 | 0.0659 | 9 |
| 20.3 | 32.9357 | 48 | 29.5294 | 63.3411 | 19.9224 | 0.085 | 4 | 0.0656 | 3 | 0.0492 | 9 |
| 21 | 36.2883 | 60 | 29.6763 | 58.961 | 16.884 | 0.0569 | 4 | 0.058 | 5 | 0.0725 | 6 |
| 23 | 34.3298 | 81 | 29.6947 | 70.6612 | 14.8153 | 0.0485 | 1 | 0.011 | 4 | 0.044 | 6 |
| 23 | 36.3939 | 40 | 29.9592 | 85.6815 | 16.4735 | 0.0604 | 3 | 0.0566 | 4 | 0.0755 | 7 |
| 24 | 32.2322 | 53 | 29.7567 | 67.9212 | 12.9284 | 0.0595 | 3 | 0.0517 | 5 | 0.0862 | 0 |
| 24 | 33.5836 | 54 | 29.757 | 63.3011 | 18.3699 | 0.0474 | 0 | 0 | 6 | 0.0984 | 5 |
| 25 | 36.7563 | 78 | 29.8049 | 99.5819 | 13.9156 | 0.0485 | 1 | 0.0114 | 4 | 0.0455 | 6 |
| 25 | 34.9043 | 72 | 29.5962 | 75.7013 | 9.9119 | 0.0547 | 5 | 0.0633 | 4 | 0.0506 | 3 |
| 25 | 33.5836 | 42 | 29.757 | 55.2209 | 21.1957 | 0.0604 | 2 | 0.0364 | 6 | 0.1091 | 7 |
| 25 | 36.157 | 82 | 29.9592 | 94.7018 | 9.2693 | 0.0485 | 2 | 0.0217 | 4 | 0.0435 | 6 |
| 25 | 39.164 | 58 | 30.0326 | 83.0015 | 21.9904 | 0.0842 | 3 | 0.0411 | 6 | 0.0822 | 9 |
| 25 | 38.6494 | 54 | 31.5781 | 67.5811 | 18.3699 | 0.0474 | 0 | 0 | 6 | 0.0984 | 5 |
| 26 | 36.4617 | 65 | 29.2025 | 84.1615 | 25.8074 | 0.0608 | 5 | 0.0649 | 5 | 0.0649 | 7 |
| 27 | 38.4853 | 67 | 30.6819 | 77.0414 | 30.4782 | 0.0842 | 4 | 0.0506 | 7 | 0.0886 | 6 |
| 27 | 34.3401 | 67 | 29.4674 | 47.5807 | 31.479 | 0.0842 | 4 | 0.0506 | 7 | 0.0886 | 6 |
| 27 | 39.2478 | 51 | 30.8964 | 74.7613 | 22.9874 | 0.0842 | 3 | 0.0448 | 4 | 0.0597 | 9 |
| 27 | 38.4853 | 51 | 30.6819 | 84.5815 | 14.6798 | 0.0531 | 5 | 0.082 | 3 | 0.0492 | 6 |
| 29 | 36.9855 | 85 | 29.8049 | 97.1018 | 8.4125 | 0.0485 | 2 | 0.0211 | 4 | 0.0421 | 6 |
| 29 | 28.7236 | 36 | 29.2648 | 41.4206 | 12.8652 | 0.035 | 4 | 0.0952 | 1 | 0.0238 | 4 |
| 30 | 39.5325 | 58 | 29.8785 | 75.3813 | 16.7932 | 0.0842 | 5 | 0.0685 | 5 | 0.0685 | 9 |
| 30 | 36.0682 | 56 | 29.612 | 87.3016 | 19.906 | 0.0995 | 5 | 0.0725 | 4 | 0.058 | 6 |
| 30 | 37.4599 | 65 | 31.5781 | 76.1613 | 11.9452 | 0.0542 | 4 | 0.0519 | 4 | 0.0519 | 6 |
| 30.7 | 33.8418 | 50 | 29.9014 | 64.3011 | 11.2933 | 0.0343 | 2 | 0.0357 | 6 | 0.1071 | 2 |
| 32.5 | 38.9084 | 55 | 29.9229 | 68.6812 | 16.6185 | 0.0842 | 4 | 0.0571 | 4 | 0.0571 | 9 |
| 33 | 32.4647 | 59 | 28.7242 | 61.301 | 6.8469 | 0.0348 | 3 | 0.0462 | 2 | 0.0308 | 6 |
| 33 | 35.7222 | 69 | 30.6645 | 64.0811 | 10.1877 | 0.0317 | 1 | 0.0135 | 3 | 0.0405 | 3 |
| 33 | 30.8143 | 42 | 29.9592 | 56.0009 | 6.0007 | 0.0386 | 1 | 0.0204 | 3 | 0.0612 | 4 |
| 33 | 34.4766 | 61 | 28.9977 | 76.9614 | 13.3347 | 0.0545 | 5 | 0.0758 | 4 | 0.0606 | 3 |
| 33 | 32.3619 | 60 | 29.9592 | 60.521 | 15.7983 | 0.058 | 3 | 0.0435 | 3 | 0.0435 | 6 |
| 33 | 34.4007 | 68 | 28.7242 | 63.7411 | 10.4681 | 0.0317 | 1 | 0.0137 | 4 | 0.0548 | 3 |
| 33 | 32.8065 | 49 | 29.4963 | 46.0807 | 11.8115 | 0.0566 | 1 | 0.0175 | 4 | 0.0702 | 6 |
| 33 | 34.5321 | 47 | 28.9075 | 66.9411 | 9.7007 | 0.0495 | 1 | 0.0185 | 4 | 0.0741 | 4 |
| 33.1 | 30.8143 | 48 | 29.9592 | 55.6609 | 8.8612 | 0.0495 | 1 | 0.0185 | 4 | 0.0741 | 2 |
| 34 | 33.9741 | 56 | 29.3772 | 69.5812 | 21.236 | 0.0562 | 2 | 0.0303 | 4 | 0.0606 | 5 |
| 34 | 32.3183 | 65 | 28.8169 | 53.4408 | 6.6755 | 0.0381 | 3 | 0.0385 | 3 | 0.0385 | 9 |
| 34 | 36.1421 | 40 | 29.612 | 85.3615 | 16.4735 | 0.0604 | 3 | 0.0566 | 4 | 0.0755 | 7 |
| 34 | 34.8762 | 33 | 28.9215 | 54.8209 | 24.8322 | 0.0562 | 4 | 0.0952 | 3 | 0.0714 | 3 |
| 35 | 36.9785 | 85 | 29.8049 | 95.5618 | 16.7962 | 0.0485 | 2 | 0.0211 | 4 | 0.0421 | 6 |
| 36 | 36.3939 | 55 | 29.9592 | 92.9217 | 19.0573 | 0.0559 | 3 | 0.0429 | 5 | 0.0714 | 9 |
| 36 | 33.3672 | 36 | 29.4191 | 70.6812 | 31.9898 | 0.0562 | 4 | 0.0889 | 5 | 0.1111 | 3 |
| 36 | 38.9248 | 51 | 31.2366 | 75.4813 | 22.2515 | 0.0842 | 3 | 0.0448 | 4 | 0.0597 | 9 |
| 36 | 36.3729 | 38 | 29.7567 | 70.6612 | 7.1823 | 0.0596 | 4 | 0.0741 | 2 | 0.037 | 9 |
| 36 | 32.4038 | 81 | 29.6763 | 55.2009 | 11.8217 | 0.0485 | 1 | 0.011 | 4 | 0.044 | 6 |
| 36 | 38.9248 | 51 | 31.2366 | 69.0012 | 22.5928 | 0.0842 | 3 | 0.0448 | 4 | 0.0597 | 9 |
| 37 | 38.5035 | 73 | 29.6763 | 77.6014 | 21.1989 | 0.0536 | 4 | 0.0476 | 5 | 0.0595 | 7 |
| 37 | 35.5614 | 71 | 28.3833 | 62.8211 | 5.8031 | 0.0976 | 2 | 0.026 | 2 | 0.026 | 6 |
| 37 | 37.0643 | 76 | 30.0254 | 86.0016 | 23.8009 | 0.065 | 1 | 0.0114 | 6 | 0.0682 | 9 |
| 37 | 36.1805 | 73 | 29.7278 | 83.0015 | 23.4643 | 0.061 | 4 | 0.0476 | 6 | 0.0714 | 6 |
| 37.3 | 41.7241 | 68 | 30.199 | 78.7214 | 12.6985 | 0.0311 | 2 | 0.0238 | 3 | 0.0357 | 9 |
| 38 | 36.8482 | 54 | 29.223 | 92.4417 | 22.7373 | 0.0842 | 3 | 0.0429 | 6 | 0.0857 | 9 |
| 38 | 39.4491 | 55 | 30.8964 | 80.6014 | 22.3174 | 0.0842 | 3 | 0.0429 | 4 | 0.0571 | 9 |
| 38 | 38.6318 | 68 | 30.2724 | 71.5612 | 31.2914 | 0.0741 | 3 | 0.0395 | 7 | 0.0921 | 6 |
| 38.3 | 37.8074 | 65 | 30.8457 | 83.8415 | 18.4392 | 0.0842 | 4 | 0.0526 | 4 | 0.0526 | 6 |
| 38.6 | 38.3691 | 51 | 30.1746 | 81.4615 | 17.9529 | 0.0842 | 4 | 0.0597 | 4 | 0.0597 | 9 |
| 39.8 | 31.174 | 42 | 29.9592 | 47.6407 | 6.2225 | 0.0357 | 1 | 0.0208 | 4 | 0.0833 | 3 |
| 40 | 35.6723 | 49 | 28.4247 | 65.5211 | 16.9431 | 0.072 | 3 | 0.0517 | 4 | 0.069 | 6 |
| 40 | 31.8422 | 47 | 29.612 | 52.3608 | 19.98 | 0.0604 | 4 | 0.0741 | 4 | 0.0741 | 3 |
| 40.5 | 38.5797 | 54 | 30.1746 | 76.9813 | 18.3052 | 0.0842 | 4 | 0.0571 | 4 | 0.0571 | 9 |
| 41 | 37.3479 | 27 | 30.9276 | 80.9614 | 8.6444 | 0.0871 | 2 | 0.069 | 1 | 0.0345 | 0 |
| 41 | 38.2737 | 68 | 30.6819 | 69.6212 | 31.8955 | 0.0842 | 4 | 0.0494 | 7 | 0.0864 | 6 |
| 41 | 25.9663 | 44 | 29.4191 | 29.9003 | 13.3785 | 0.085 | 4 | 0.0727 | 3 | 0.0545 | 6 |
| 42 | 37.1691 | 75 | 30.6645 | 81.8415 | 11.5261 | 0.0545 | 5 | 0.0633 | 4 | 0.0506 | 3 |
| 42.9 | 31.6737 | 41 | 29.4918 | 46.7807 | 7.5819 | 0.0358 | 1 | 0.0208 | 4 | 0.0833 | 3 |
| 43 | 34.4598 | 37 | 29.612 | 81.7015 | 24.248 | 0.0562 | 5 | 0.1087 | 3 | 0.0652 | 3 |
| 43 | 31.6842 | 76 | 29.4674 | 56.9609 | 2.8908 | 0.0976 | 2 | 0.0241 | 2 | 0.0241 | 6 |
| 44 | 35.7771 | 68 | 29.5477 | 55.0809 | 10.4781 | 0.0545 | 5 | 0.0658 | 4 | 0.0526 | 6 |
| 44 | 37.8407 | 77 | 30.3454 | 75.2413 | 9.9991 | 0.0976 | 2 | 0.0233 | 4 | 0.0465 | 6 |
| 44 | 34.8319 | 61 | 29.6763 | 66.6611 | 15.9663 | 0.0394 | 5 | 0.0714 | 4 | 0.0571 | 6 |
| 44 | 35.912 | 61 | 29.4263 | 78.6414 | 21.7026 | 0.0586 | 4 | 0.0556 | 5 | 0.0694 | 6 |
| 46 | 36.005 | 64 | 30.6645 | 69.5612 | 12.475 | 0.0545 | 5 | 0.0725 | 4 | 0.058 | 3 |
| 47 | 35.8572 | 57 | 30.8457 | 79.5214 | 23.6031 | 0.0559 | 4 | 0.0556 | 5 | 0.0694 | 9 |
| 47 | 37.3479 | 55 | 30.9276 | 84.7615 | 16.4942 | 0.085 | 4 | 0.0606 | 4 | 0.0606 | 6 |
| 47 | 35.8572 | 55 | 30.8457 | 68.7412 | 16.4942 | 0.085 | 4 | 0.0606 | 4 | 0.0606 | 6 |
| 47 | 37.1226 | 44 | 30.7802 | 87.9016 | 13.3785 | 0.085 | 4 | 0.0727 | 3 | 0.0545 | 6 |
| 48 | 33.2528 | 42 | 29.4191 | 51.3408 | 1.1035 | 0.0532 | 4 | 0.08 | 2 | 0.04 | 6 |
| 49 | 33.9166 | 59 | 29.339 | 77.0614 | 13.866 | 0.0394 | 5 | 0.0735 | 3 | 0.0441 | 6 |
| 49 | 35.4079 | 53 | 29.4674 | 51.6808 | 28.3591 | 0.084 | 4 | 0.0606 | 4 | 0.0606 | 7 |
| 50 | 32.6268 | 41 | 29.86 | 54.2609 | 10.7224 | 0.0558 | 2 | 0.0417 | 5 | 0.1042 | 4 |
| 50 | 34.5641 | 68 | 29.6947 | 92.5617 | 17.0342 | 0.0579 | 3 | 0.039 | 4 | 0.0519 | 7 |
| 50 | 36.0959 | 51 | 29.3772 | 79.2014 | 25.1575 | 0.0604 | 3 | 0.0484 | 5 | 0.0806 | 6 |
| 50 | 33.621 | 90 | 29.7567 | 53.1008 | 13.3877 | 0.0569 | 3 | 0.0323 | 4 | 0.043 | 3 |
| 51 | 36.7425 | 66 | 31.1982 | 77.3014 | 12.4484 | 0.0323 | 3 | 0.0366 | 3 | 0.0366 | 10 |
| 51 | 36.7425 | 34 | 31.1982 | 64.0611 | 19.38 | 0.0561 | 4 | 0.093 | 2 | 0.0465 | 3 |
| 51 | 38.9963 | 40 | 31.1982 | 79.2614 | 13.1589 | 0.0842 | 3 | 0.0588 | 3 | 0.0588 | 6 |
| 51 | 36.6037 | 40 | 30.5162 | 73.6213 | 13.1589 | 0.0842 | 3 | 0.0588 | 3 | 0.0588 | 6 |
| 51 | 32.2109 | 65 | 28.6643 | 48.9007 | 16.32 | 0.0545 | 6 | 0.0845 | 4 | 0.0563 | 3 |
| 53 | 39.7799 | 56 | 30.7531 | 75.8213 | 22.6414 | 0.0842 | 4 | 0.0563 | 4 | 0.0563 | 9 |
| 53 | 33.6981 | 53 | 29.2325 | 61.821 | 9.9896 | 0.0565 | 4 | 0.0656 | 3 | 0.0492 | 6 |
| 53.1 | 33.0526 | 46 | 30.0254 | 51.5408 | 16.6163 | 0.0529 | 1 | 0.0192 | 6 | 0.1154 | 2 |
| 54 | 40.0039 | 57 | 31.1155 | 85.9016 | 17.5532 | 0.0546 | 3 | 0.0448 | 3 | 0.0448 | 6 |
| 54 | 31.4518 | 75 | 29.6665 | 46.7407 | 10.2769 | 0.0545 | 7 | 0.0864 | 5 | 0.0617 | 3 |
| 54 | 33.5926 | 57 | 29.612 | 52.6208 | 17.5532 | 0.0546 | 3 | 0.0448 | 3 | 0.0448 | 6 |
| 54 | 33.5926 | 53 | 29.612 | 63.3211 | 17.003 | 0.0842 | 4 | 0.058 | 5 | 0.0725 | 9 |
| 54 | 38.6662 | 26 | 30.1759 | 75.3013 | 22.8104 | 0.0396 | 1 | 0.0278 | 6 | 0.1667 | 3 |
| 59 | 35.9383 | 44 | 30.6214 | 60.001 | 11.5534 | 0.0554 | 2 | 0.0392 | 5 | 0.098 | 3 |
| 62 | 37.235 | 59 | 30.7802 | 68.2411 | 17.396 | 0.0842 | 4 | 0.0563 | 5 | 0.0704 | 6 |
| 63 | 37.1957 | 80 | 29.9978 | 95.6618 | 17.388 | 0.0516 | 1 | 0.0109 | 5 | 0.0543 | 9 |
| 64 | 37.9417 | 72 | 30.3926 | 71.9012 | 17.604 | 0.0569 | 4 | 0.0471 | 5 | 0.0588 | 9 |
| 64 | 37.1256 | 68 | 29.6763 | 75.1013 | 15.507 | 0.0545 | 7 | 0.0933 | 4 | 0.0533 | 3 |
| 65 | 37.6513 | 69 | 30.5855 | 76.8614 | 6.252 | 0.038 | 2 | 0.027 | 4 | 0.0541 | 3 |
| 65 | 34.934 | 67 | 28.9215 | 63.5211 | 18.0354 | 0.0545 | 5 | 0.0676 | 6 | 0.0811 | 3 |
| 66 | 38.7472 | 76 | 29.6763 | 82.8215 | 19.6978 | 0.0525 | 4 | 0.046 | 5 | 0.0575 | 7 |
| 66 | 34.0727 | 42 | 29.9337 | 48.0207 | 13.2051 | 0.0971 | 3 | 0.0588 | 1 | 0.0196 | 6 |
| 68 | 31.2565 | 35 | 29.364 | 51.5208 | 18.9854 | 0.0561 | 5 | 0.1136 | 2 | 0.0455 | 3 |
| 69 | 34.962 | 42 | 29.7714 | 74.9613 | 32.036 | 0.0604 | 1 | 0.0182 | 8 | 0.1455 | 7 |
| 69 | 37.7893 | 59 | 30.5487 | 54.6409 | 7.3948 | 0.0394 | 6 | 0.087 | 3 | 0.0435 | 6 |
| 70 | 36.2323 | 37 | 31.7169 | 58.341 | 16.2109 | 0.0757 | 5 | 0.1042 | 3 | 0.0625 | 3 |
| 71 | 31.4066 | 54 | 29.7278 | 52.5808 | 8.2904 | 0.0547 | 2 | 0.0345 | 3 | 0.0517 | 3 |
| 72 | 36.4796 | 70 | 30.6645 | 72.4013 | 11.3715 | 0.0545 | 5 | 0.0667 | 4 | 0.0533 | 3 |
| 72 | 34.3503 | 46 | 29.2648 | 79.0214 | 8.4852 | 0.0989 | 4 | 0.0645 | 1 | 0.0161 | 10 |
| 73 | 35.8102 | 47 | 30.8318 | 85.4416 | 32.1986 | 0.0979 | 3 | 0.0508 | 7 | 0.1186 | 7 |
| 75.1 | 35.9046 | 54 | 30.9276 | 70.8212 | 9.6085 | 0.0546 | 4 | 0.0615 | 3 | 0.0462 | 6 |
| 76 | 35.8031 | 60 | 30.5487 | 69.4212 | 9.9537 | 0.0545 | 6 | 0.0923 | 3 | 0.0462 | 3 |
| 77 | 28.5219 | 37 | 29.7278 | 41.2606 | 9.8622 | 0.0568 | 2 | 0.0488 | 3 | 0.0732 | 3 |
| 77 | 37.068 | 65 | 28.8701 | 78.8014 | 23.4306 | 0.0586 | 4 | 0.0541 | 6 | 0.0811 | 3 |
| 78 | 33.3015 | 49 | 28.771 | 45.9207 | 16.6451 | 0.0605 | 3 | 0.0545 | 4 | 0.0727 | 4 |
| 82 | 39.254 | 71 | 31.1866 | 69.5012 | 11.6019 | 0.0545 | 7 | 0.0897 | 4 | 0.0513 | 3 |
| 84.2 | 32.8541 | 50 | 29.364 | 52.6808 | 4.9739 | 0.0546 | 5 | 0.0862 | 2 | 0.0345 | 6 |
| 85 | 30.1039 | 40 | 29.7278 | 48.0607 | 9.5151 | 0.0559 | 2 | 0.0426 | 3 | 0.0638 | 3 |
| 85 | 29.6006 | 39 | 29.9014 | 46.7207 | 8.2182 | 0.0529 | 1 | 0.0217 | 3 | 0.0652 | 6 |
| 86 | 38.406 | 71 | 30.6645 | 86.9416 | 9.0759 | 0.0712 | 5 | 0.061 | 4 | 0.0488 | 6 |
| 87 | 35.6405 | 62 | 30.7171 | 64.421 | 8.0755 | 0.0545 | 5 | 0.0746 | 3 | 0.0448 | 3 |
| 88.5 | 42.1592 | 88 | 31.1866 | 110.4621 | 12.1122 | 0.0562 | 4 | 0.0392 | 4 | 0.0392 | 9 |
| 89 | 33.2217 | 56 | 29.1987 | 69.8212 | 21.7432 | 0.0399 | 1 | 0.0152 | 7 | 0.1061 | 6 |
| 89 | 35.3181 | 36 | 30.046 | 66.2811 | 11.6121 | 0.0992 | 6 | 0.12 | 1 | 0.02 | 9 |
| 90 | 38.8452 | 33 | 31.1866 | 73.6613 | 3.022 | 0.0439 | 2 | 0.0465 | 3 | 0.0698 | 6 |
| 90 | 34.776 | 65 | 31.559 | 55.8609 | 11.0351 | 0.0545 | 7 | 0.0972 | 4 | 0.0556 | 3 |
| 92 | 35.8194 | 67 | 29.6947 | 66.0211 | 5.3725 | 0.038 | 3 | 0.0411 | 4 | 0.0548 | 3 |
| 94 | 36.2438 | 67 | 30.6645 | 69.2212 | 10.9875 | 0.0545 | 5 | 0.0694 | 4 | 0.0556 | 3 |
| 96 | 37.1475 | 72 | 29.4963 | 71.0612 | 5.7945 | 0.0394 | 6 | 0.0741 | 4 | 0.0494 | 5 |
| 97 | 36.8412 | 40 | 30.6214 | 77.6614 | 8.6386 | 0.0718 | 5 | 0.0893 | 5 | 0.0893 | 6 |
| 98 | 30.7669 | 50 | 28.7929 | 54.0209 | 13.805 | 0.0881 | 2 | 0.037 | 2 | 0.037 | 3 |
| 100 | 34.3391 | 61 | 29.9337 | 45.0406 | 9.6373 | 0.0394 | 6 | 0.0845 | 4 | 0.0563 | 6 |
| 100 | 35.5407 | 44 | 29.4676 | 75.4213 | 12.1892 | 0.0971 | 3 | 0.0556 | 1 | 0.0185 | 6 |
| 100 | 38.114 | 51 | 30.5162 | 50.2208 | 11.4386 | 0.0691 | 3 | 0.0455 | 5 | 0.0758 | 9 |
| 100 | 36.1246 | 68 | 30.7171 | 71.3412 | 9.962 | 0.0545 | 5 | 0.0685 | 3 | 0.0411 | 3 |
| 100 | 37.6613 | 58 | 29.8049 | 67.1211 | 19.7031 | 0.0599 | 3 | 0.0411 | 6 | 0.0822 | 6 |
| 0 | 29.7476 | 38 | 29.9592 | 39.9805 | 11.1941 | 0.1019 | 1 | 0.0222 | 4 | 0.0889 | 3 |
| 1 | 35.4908 | 74 | 29.6763 | 71.6413 | 14.1248 | 0.0597 | 4 | 0.0465 | 5 | 0.0581 | 6 |
| 1.6 | 33.5516 | 53 | 29.3772 | 67.8411 | 24.2433 | 0.0839 | 3 | 0.0462 | 4 | 0.0615 | 6 |
| 2 | 34.3298 | 40 | 29.6947 | 77.9814 | 15.7244 | 0.0604 | 3 | 0.0566 | 4 | 0.0755 | 6 |
| 2 | 33.0241 | 44 | 29.5477 | 58.441 | 23.5789 | 0.0562 | 5 | 0.0962 | 4 | 0.0769 | 3 |
| 3 | 37.1635 | 77 | 29.0459 | 94.5617 | 15.3234 | 0.0586 | 4 | 0.046 | 5 | 0.0575 | 6 |
| 3.4 | 33.9768 | 54 | 29.339 | 69.8212 | 26.1077 | 0.0995 | 4 | 0.0606 | 4 | 0.0606 | 6 |
| 4 | 36.3443 | 61 | 29.2785 | 80.5814 | 24.7135 | 0.061 | 5 | 0.0685 | 6 | 0.0822 | 7 |
| 4 | 37.0397 | 86 | 29.7278 | 81.5215 | 11.9513 | 0.0485 | 1 | 0.0103 | 4 | 0.0412 | 6 |
| 4 | 37.6003 | 61 | 29.9592 | 97.2418 | 19.3977 | 0.0599 | 4 | 0.0548 | 6 | 0.0822 | 3 |
